# Dynamics of long-distance signaling via plant vascular tissues

**DOI:** 10.3389/fpls.2015.00161

**Published:** 2015-03-18

**Authors:** Michitaka Notaguchi, Satoru Okamoto

**Affiliations:** ^1^Graduate School of Science, Nagoya University, NagoyaJapan; ^2^ERATO Higashiyama Live-Holonics Project, NagoyaJapan; ^3^Research Fellow of the Japan Society for the Promotion of Science, TokyoJapan

**Keywords:** long-distance signaling, RNA transport, peptide transport, phloem transport, protein transport, systemic signaling, xylem transport

## Abstract

Plant vascular systems are constructed by specific cell wall modifications through which cells are highly specialized to make conduits for water and nutrients. Xylem vessels are formed by thickened cell walls that remain after programmed cell death, and serve as water conduits from the root to the shoot. In contrast, phloem tissues consist of a complex of living cells, including sieve tube elements and their neighboring companion cells, and translocate photosynthetic assimilates from mature leaves to developing young tissues. Intensive studies on the content of vascular flow fluids have unveiled that plant vascular tissues transport various types of gene product, and the transport of some provides the molecular basis for the long-distance communications. Analysis of xylem sap has demonstrated the presence of proteins in the xylem transpiration stream. Recent studies have revealed that CLE and CEP peptides secreted in the roots are transported to above ground via the xylem in response to plant–microbe interaction and soil nitrogen starvation, respectively. Their leucine-rich repeat transmembrane receptors localized in the shoot phloem are required for relaying the signal from the shoot to the root. These findings well-fit to the current scenario of root-to-shoot-to-root feedback signaling, where peptide transport achieves the root-to-shoot signaling, the first half of the signaling process. Meanwhile, it is now well-evidenced that proteins and a range of RNAs are transported via the phloem translocation system, and some of those can exert their physiological functions at their destinations, including roots. Thus, plant vascular systems may serve not only as conduits for the translocation of essential substances but also as long-distance communication pathways that allow plants to adapt to changes in internal and external environments at the whole plant level.

## Introduction

Plant vascular tissues are formed through highly specialized cell wall modifications to achieve their roles as conduits of water and nutrients. The processes of cell differentiation in xylem and phloem tissues have been intensively studied, and many regulatory genes have been identified and characterized (only relatively new topics are introduced in this review). Through the functions of such genetic components, xylem vessels, and phloem sieve tubes are formed running through the entire plant body. Such structures are well-designed to play their roles of conduits for water and nutrients.

Another facet of plant vascular tissues has been also described, that is their use as long-distance signaling pathways (Lucas et al., 2013). For multicellular organisms, communication at the entire body level is an essential task to coordinate behaviors within the organism as a singular living being. Whereas animals have evolved nervous systems to transmit information signal from one part of body to another, plants do not have a nervous system. Plants do use electrical signals to transmit information, for example, soon after wounding (Mousavi et al., 2013) or after salt stress treatment (Choi et al., 2014). However, electrical systems in plants appear not to be sufficient to provide the basis for expansive systemic responses. Alternatively, plants seem to use vascular tissues for long-distance communication. This review summarizes the potential signal agents transported via xylem and phloem, and especially focuses on gene-encoded macromolecules. Together, we discuss on the bi-directional flow of the information and the circuits used for signaling through these conduit tissues.

## Xylem Signaling

Xylem consists of TEs, parenchyma cells, and fiber cells. These cells become differentiated from derivatives of the apical meristems (Evert, 2006), and dedicated studies have identified many factors involved in xylem cell differentiation and patterning (Schuetz et al., 2013; Kondo et al., 2014). TEs are formed as shells of cells that possess thickened secondary cell walls and lose their nuclei and cell contents through programmed cell death (Fukuda, 1996). Multiple TEs compose conduits, and continuous conduits connect various organs and tissues from the root to the shoot. Xylem vessels provide physical support for aerial organs and transport water and essential nutrients from the soil. In addition, xylem transports various molecules including long-distance signaling factors that mediate organ-to-organ communication.

*Trans*-zeatin type CKs have been mainly detected in xylem sap (Takei et al., 2001). Grafting experiments using a quadruple mutant of CK synthetic genes, *ATP/ADP IPT 1*; *3*; *5*; *7*, indicated that a *trans*-zeatin type of CKs is transported from the roots to the shoots and regulate shoot growth (Matsumoto-Kitano et al., 2008). An ABC transporter, ABCG14, has been suggested to play a role in loading CKs to xylem (Ko et al., 2014; Zhang et al., 2014). *ABCG14* is primary expressed in root vascular tissues, and the defect in *ABCG14* resulted in an accumulation of CKs in roots. Strigolactones (SLs) control shoot branching as well as known as root-secreted signals for interactions with symbiotic fungi and parasitic weeds (Bouwmeester et al., 2003; Akiyama et al., 2005; Gomez-Roldan et al., 2008; Umehara et al., 2008). In inhibition of shoot branching, SLs and their precursor, carlactone, were proposed to be long-distance signaling factors. SLs have been detected in xylem sap (Kohlen et al., 2011), whereas grafting experiments using a series of mutants of SL synthetic enzymes and biochemical analyses on SL synthetic pathway suggested that carlactone is a root-to-shoot mobile signal (Booker et al., 2005; Seto and Yamaguchi, 2014; Seto et al., 2014). Although the major player in long-distance inhibition of shoot branching is still unknown, these findings describe that small phytohormones play essential roles in plant root-to-shoot coordination.

### Xylem Mobile Proteins

In addition to phytohormones, macromolecules, such as proteins, were detected from xylem exudates in Biles and Abeles (1991) and Satoh et al. (1992). Since that time, many proteins have been identified in xylem sap of various plant species including *Cucumis sativus, Brassica oleracea, Zea mays,* and *Glycine max* (Sakuta et al., 1998; Masuda et al., 1999; Rep et al., 2002; Buhtz et al., 2004; Kehr et al., 2005; Djordjevic et al., 2007; Aki et al., 2008; Alvarez et al., 2008; Fernandez-Garcia et al., 2011; Ligat et al., 2011). Xylem sap is easy to collect from those large-sized plants with root pressure, and many proteins have been identified in the sap, including structural proteins of cell walls and defense-related proteins. Molecular genetic approach has been applied to XSP10 in tomato. XSP10 is a cysteine-rich 10 kDa secreted protein and displays structural similarity to lipid transfer protein (Rep et al., 2003). *XSP10* is expressed in roots and lower stems. By using *XSP10*-silenced tomato, Krasikov et al. (2011) reported that XSP10 is involved in the susceptibility of tomato to a fungal vascular pathogen. Although using molecular genetics approaches on those non-model plants is not easy, functional analyses of proteins associated with xylem sap may reveal the roles of xylem in many aspects of plant life.

### Xylem Mobile Small Peptides

**FIGURE 1:**
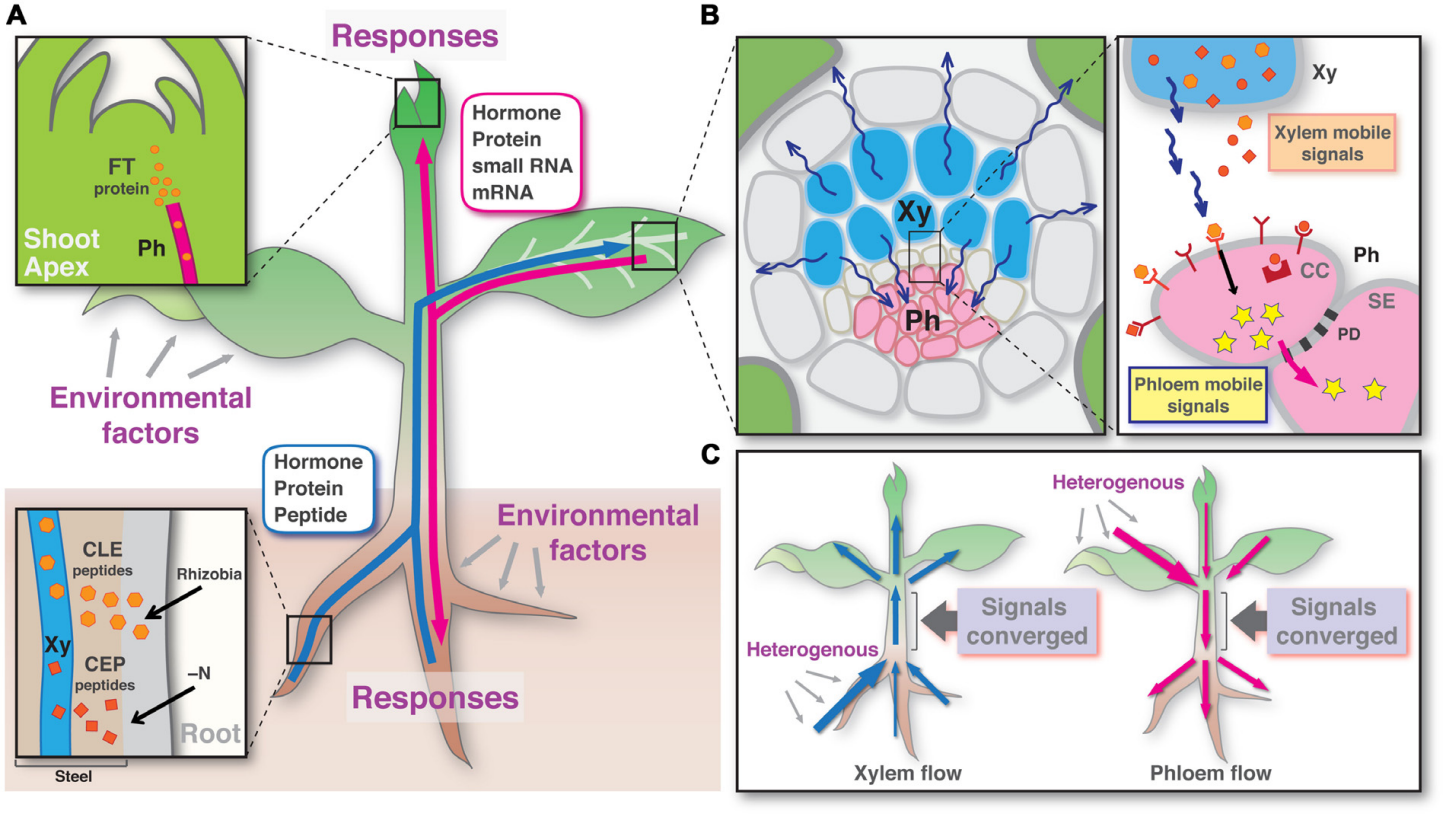
**A model of long-distance signaling via plant vascular tissues. (A)** Potential signal molecules of the xylem (blue) and the phloem (red) translocation pathways. Insets show xylem loading and phloem unloading of signal molecules in the sink tissues. **(B)** Signal relay from the xylem to the phloem in the leaf vein. **(C)** Signal convergence by running through a stem region in each of xylem and phloem pathways. Xy, xylem; Ph, phloem; CC, companion cell; SE, sieve element; PD, plasmodesmata.

As genomic information becomes available, many genes that encode small-secreted peptide have been found in various plant species. It is predicted that *Arabidopsis* genome contains more than 900 peptide genes (Matsubayashi, 2011). Intensive studies on some of these peptides and their receptors have revealed that a number of secreted peptides play an important role in relatively short-range cell-to-cell communication (Fletcher et al., 1999; Hirakawa et al., 2008; Ohyama et al., 2009; Lee et al., 2012). The CLV3/CLV1 ligand/receptor pair is a well-known cell-to-cell signaling model, where its active form of CLV3 peptide belonging to the CLE family is perceived by CLV1 LRR-RK (Ohyama et al., 2009). *CLV3* and *CLV1* are expressed in adjacent cells in the shoot apex and control the activity of the shoot apical meristem in same genetic pathway (Clark et al., 1995, 1997; Fletcher et al., 1999). On the other hand, in xylem that is a kind of apoplast, whether small-secreted peptides exist and mediate organ-to-organ communication remained unknown. Recently, secreted oligopeptides belonging to the CLE peptide or the CEP family have been shown to be translocated from the roots to the shoots to act as long-distance signaling factors in systemic suppression of nodule formation or in nitrogen starvation response of root systems, respectively (Okamoto et al., 2013; Tabata et al., 2014; **Figure 1A**). We summarize recent findings related to those two secreted peptides below.

Leguminous plants establish a symbiosis with soil bacteria, called rhizobia, and form nodules on their roots. Because excessive nodule formation is harmful to host plants, the plants control the number of nodules via a root-to-shoot-to-root long-distance feedback loop (Caetano-Anolles and Gresshoff, 1991). This regulatory loop is called autoregulation of nodulation, and two long-distance signals, namely, “root-derived signal (root-to-shoot)” and “shoot-derived inhibitor (shoot-to-root)” have been postulated. However, the entities of those long-distance signals have been unknown for more than two decades. As a strong candidate for the root-derived signal, CLE-RS2 oligopeptide was identified from a model legume *Lotus japonicus*. *CLE-RS2* is expressed in roots and is highly up regulated by rhizobial inoculation (Okamoto et al., 2009). The active form of CLE-RS2 is a glycosylated 13-amino acid oligopeptide, and it strongly suppressed nodule formation. Importantly, biochemical analyses have revealed that CLE-RS2 glycopeptide was translocated from the root to the shoot via xylem, and that it directly bound HAR1 receptor that is a CLV1-homologous receptor and a shoot factor for the autoregulation of nodulation (Krusell et al., 2002; Nishimura et al., 2002; Okamoto et al., 2013). Homologs of *CLE-RS2* and *HAR1* were found in *Medicago truncatula* and soybean, suggesting that this signaling mechanism is common in legumes (Nishimura et al., 2002; Searle et al., 2003; Schnabel et al., 2005; Mortier et al., 2010, 2011; Lim et al., 2011; Reid et al., 2011).

Systemic signaling of CLE-RS2/HAR1 were implied in the studies on nitrate inhibition of nodulation, where *CLE-RS2* was also strongly up regulated under high nitrate conditions that are known to abolish nodulation (Okamoto et al., 2009) and grafting experiments using a *har1* mutant showed that shoot-expressed HAR1 is critical to nitrate inhibition of nodulation (Okamoto and Kawaguchi, 2015). Additionally, root overexpression of two types of *CLE-RS2* homologs suppressed nodulation in systemic or local manner in soybean (Reid et al., 2011). Domain-swapping experiments between them showed that not only CLE domain but also the external regions are important for the activity to suppress nodulation (Reid et al., 2013). This could reflect the difference of each CLE peptides in their activation or recognition by their receptors. In *Arabidopsis, CLE6* overexpression in roots affected shoot morphology (Bidadi et al., 2014). Thus, it appears that CLE peptide-mediated long-distance signaling is not specific to legumes.

Another family of secreted peptides, CEPs, is involved in root-to-shoot signaling triggered by nitrogen starvation. CEP peptides were identified using an *in silico* approach on the *Arabidopsis* genome. Mature CEP1 peptide is a 15 aa oligopeptide that is derived from a conserved domain of the C-terminal region of precursor polypeptide (Ohyama et al., 2008). It is reported that some *CEP* genes responds to the nitrogen-poor conditions (Delay et al., 2013; Tabata et al., 2014). Tabata et al. (2014) identified CEP1 receptors in *Arabidopsis*, XIP1/CEPR1 and CEPR2, and found that a double mutant of *CEPR*s exhibited a pleiotropic phenotype relative to nitrogen starvation. Application of CEP1 peptides into roots resulted in up-regulation of *NRT2.1*, encoding a nitrate transporter, in a shoot CEPRs-dependent manner, and some CEP peptides have been detected in the xylem sap. It is proposed that plants employ several types of nitrogen-related systemic root-to-shoot-to-root signaling and coordinate root responses in heterogeneous nitrogen environments at the whole plant level (Ruffel et al., 2011). These results strongly suggest that CEP peptide functions as a nitrogen-demand root-to-shoot signal.

*CEP* genes are widespread among seed plants (Roberts et al., 2013). In *M. truncatula*, some *CEP* genes were also induced by low nitrogen conditions. Overexpression of *MtCEP1* resulted in the inhibition of lateral root formation and the enhancement of nodule formation (Imin et al., 2013). Additionally, a homolog of *CEPR1* receptor gene, *CRA2*, was also characterized in *M. truncatula*. The *cra2* mutants formed an increased number of lateral roots and a decreased number of nodules, the phenotype of which is opposite to *MtCEP1* overexpressing plants. Interestingly, grafting experiments showed that *CRA2* functions using two different pathways. *CRA2* inhibits lateral root development in a local manner, but enhances nodule formation in a systemic manner from the shoot. These findings raise the possibility that *MtCEPs* acts in both systemically and locally via *CRA2* (Huault et al., 2014).

As shown here, the studies in recent years on the secreted oligopeptides provided new insight into organ-to-organ signaling mechanism through xylem. Furthermore, a number of undetermined mobile peptides have been identified through the analysis of xylem sap in soybean (Okamoto et al., unpublished data). In addition, many receptors have been found from the transcriptomic analyses of phloem tissues (e.g., Deeken et al., 2008). These imply general importance of secreted peptide transport via xylem on plant long-distance signaling.

## Phloem Signaling

Phloem SE cells form a transport network for long-distance allocation of photosynthates and signaling molecules (Lucas et al., 2013). Mature SE cells are developed from undifferentiated future phloem cells, called protophloem SEs, through specialized autolysis processes accompanied by enucleation (Esau, 1950; Furuta et al., 2014). The protophloem cells are sequentially generated as a line in the meristematic tissues resulting in a network of phloem cells running through the entire body. Molecular genetics studies in *Arabidopsis* revealed that ALTERED PHLOEM DEVELOPMENT, a key transcription factor regulating phloem development, is expressed in the protophloem cells and promotes phloem development (Bonke et al., 2003). This phloem differentiation, accompanied by degradation of some organelles and enucleation, is achieved through the function of *NAC45/86* downstream genes, which target a family of genes, *NAC45/86 DEPENDENT EXONUCLEASE-DOMAIN PROTEIN 1-4*, encoding proteins with nuclease domains, essential for enucleation (Furuta et al., 2014). Thus, phloem tube systems are sequentially constructed through the processes of genetically controlled cell differentiation from adjacent region to the meristem and connecting to the differentiated leaves, stems, and roots.

Translocation of molecules into SEs can be achieved by two pathways; from apoplasmic to intracellular region, or cell-to-cell symplasmic transport via the specialized secondary PD interconnecting the SEs and neighboring CC (Oparka and Turgeon, 1999). At present, it is generally agreed that phloem flow is driven by a hydrostatic pressure gradient along the tube according to Münch’s pressure-flow hypothesis, although this hypothesis has not yet been supported unequivocally (Knoblauch and Oparka, 2012). Considerable obstacles to the study of phloem cause difficulties in understanding of dynamics of living phloem tissues. While xylem sap can be easily collected as root pressure exudates, phloem sap has been collected from cut insect stylets (with a rate of several μL/h) or as exudates from incisions into stems, petioles, floral axes, or fruits with the use of chelating agents, e.g., EDTA to eliminate sieve tube blockage (Hoad, 1995). Phloem flow transports small molecules such as photosynthesized sugars, metabolites and phytohormones, as well as macromolecules such as proteins and a variety of RNA species. As shown below, a portion of these phloem-mobile molecules serve as information signals. Recent reviews describing phloem transport of each group of molecules are also available elsewhere (Kehr, 2006; Lough and Lucas, 2006; Kehr and Buhtz, 2008; Lucas et al., 2013).

### Phloem Mobile Phytohormones

Analysis of phloem exudates has provided evidence for that phloem transports several phytohormones, including auxin, CKs, abscisic acid, and gibberellins (Hoad, 1995). Jasmonic acids, salicylic acids, and/or their derivatives are also proposed as components of the phloem sap in association with defense signaling (Vlot et al., 2008). IAA and other IAA derivatives have been identified from phloem sap. AUX1, a putative auxin influx carrier expressed in higher ordered vascular tissues, as well as the other members of the *AUX1* gene family, facilitate the loading of IAA into vascular tissues and the transport of IAA from source leaves to sink tissues such as roots via the phloem (Marchant et al., 2002). iP-type CKs are main contents of leaf exudates, whereas tZ-type CKs are the major species in the xylem (Sakakibara, 2006). A biosynthesis gene, *AtIPT3*, and a purine permease gene, *AtPUP2*, are expressed in phloem tissues (Burkle et al., 2003; Miyawaki et al., 2004; Takei et al., 2004). The latter is involved in CK nucleobase uptake to retrieve it into the phloem. Thus, the transportation of auxins and CKs via the phloem appears to be a controlled process. Additionally, hormone concentrations in the phloem change in response to the environmental and developmental conditions (Hoad, 1995). Therefore, mobile hormones in phloem should have key roles in controlling the physiology of plants, although the precise nature of most of their roles and the underling mechanisms remain to be elucidated. Recently CKs were proposed as a shoot-derived inhibitor in nodulation (see the later section).

### Phloem Mobile Proteins and RNAs

The phloem translocation stream contains hundreds of proteins and hundreds of transcripts, including mRNA, small RNA, and long non-coding RNA. In the last decade, their roles in long-distance signaling have been demonstrated. The importance of protein transport has been established unequivocally by a case of FT, a florigen. FT and its homolog proteins expressing in the leaves promote meristem outgrowth, such as flowering in the shoot apical meristems (**Figure 1A**) and the initiation of growth of dormant buds or secondary shoot primordia (Böhlenius et al., 2006; Hiraoka et al., 2013; Niwa et al., 2013) as well as the tuberization in the underground stolons (Navarro et al., 2011). Meanwhile, several types of RNA species have been thought as phloem-mobile (Kehr and Buhtz, 2008), and the most agreed phenomenon involving RNA transport is siRNA triggering systemic silencing which is the basis for systemic acquired resistance (Bologna and Voinnet, 2014).

The discovery of FT protein transport, first as a florigenic signal, was led by the studies on photoperiodic flowering initiated early in the 19th century (Lang, 1965). Physiological experiments have revealed plant “phototropism” (Garner and Allard, 1920) and demonstrated the presence of hormone-like systemic signals generated in leaves under favorable photoperiodic conditions (Chailakhyan, 1937). Genetic studies on flowering have successfully identified a number of locus and/or genes related to flowering especially in pea, rice and *Arabidopsis* (Rédei, 1975; Koornneef et al., 1998; Yano et al., 2001; Weller et al., 2009). Subsequent characterization of these genes has narrowed down the position of the florigenic signal in the genetic cascades, between *FT* and *FD* genes (Abe et al., 2005; Wigge et al., 2005). Finally, the current conclusion that long-distance transport of FT protein, as a florigen, via phloem is the molecular basis for the promotion of flowering by photoperiodic control was agreed through the studies conducted by several research groups (Corbesier et al., 2007; Jaeger and Wigge, 2007; Lin et al., 2007; Mathieu et al., 2007; Tamaki et al., 2007; Notaguchi et al., 2008). An important role of FT, or its homologs, as it relates to flowering has been described in many plant species. Additionally, in chrysanthemum, *CsAFT*, a PEBP family gene which belongs to a different group from the FT group, is also generated in leaves and then moved to the apex to suppress flowering, which may be an antiflorigen, another proposed systemic signal which controls flowering (Higuchi et al., 2013). The transport mechanisms of FT or its homologs are as yet largely unknown, except for a clue from an ER membrane localized exporter, FT-INTERACTING PROTEIN 1, which is involved in protein loading of FT from the CC into the SE tube system (Liu et al., 2012). The existence of regulatory systems in long-distance FT transport has been also implied from the fact that amino acid substitutions of FT protein caused a defect only in its mobility, but not in activity that promotes flowering (Yoo et al., 2013). Near the shoot apical meristems, FT-GFP protein was detected in the provasculature at the apex and at the base of the shoot apical meristem, but this pattern was not observed in very young seedlings (Corbesier et al., 2007). These observations further support the presence of regulatory systems for FT protein movement.

Phloem exudates contain a number of proteins for the protection of functional phloem tubes. For an example, major structural phloem proteins encoded by *SE occlusion* gene family have roles in wound sealing of SEs to avoid nutrient loss (Ernst et al., 2012; Jekat et al., 2013). The analyses of phloem exudates also have identified a large quantity of proteins that are functionally related to defense response, such as proteinase inhibitors, lectins, and other proteins induced by wounding or insect feeding. These proteins disrupt feeding as well as digestion of phloem contents and includes some toxic proteins (Kehr, 2006). However, the functions of many of phloem proteins have yet to be elucidated. While the presence of a type of passive transport into SEs has been depicted by diffusion of GFP fused-proteins (Stadler et al., 2005), active transport may also occur because the phloem protein population includes proteins larger than 100 kDa which is in excess of the PD size exclusion limits and such protein fractions have ability to increase size exclusion limits (Balachandran et al., 1997). Why plants deliver only a part of their proteins is an important question to be answered to understand the function of the phloem translocation stream as well as plant systemic signaling.

Endogenous cellular RNAs are known to be transported systemically; small non-coding RNAs, such as siRNA and miRNA, and mRNAs (Kehr and Buhtz, 2008). Among these, the function of siRNA has been most well-established. The mobility of siRNA was clearly explained by grafting experiments with multiple dicer mutants as recipients in which siRNA production does not occur. siRNA movement triggers post-transcriptional gene silencing and also transcriptional gene silencing, which is crucial for the achievement of systemic acquired resistance to pathogens and viruses in plants (Dunoyer et al., 2010; Molnar et al., 2010). miRNA movement via phloem is also supported by several evidences (Marín-González and Suárez-López, 2012). miR399 has been proposed as a mobile signal in response to phosphate starved conditions (Lin et al., 2008; Pant et al., 2008). miR395 has been identified using miRNA processing mutant *hen1-1* under sulfate starved conditions (Buhtz et al., 2010). The mobility of the miR172 and miR156 regulating phase transitions such as flowering and tuberization have also been demonstrated (Martin et al., 2009; Kasai et al., 2010; Bhogale et al., 2014). Non-coding RNAs longer than si/miRNAs, ranging from 30 to 90 bases, were also found from pumpkin phloem exudates and, in the *in vitro* tests, they showed activities that inhibit protein translation (Zhang et al., 2009). Thus, non-coding RNAs regulate their target expression levels in their target tissues in their own manners.

mRNA transport has been also well-evidenced by grafting experiments (Notaguchi, 2015). Although the functions of mRNAs have been not clarified yet, their effect of several mRNA species on development has been observed; a dominant form of a *KNOX* mRNA, named *Me*, can result in altered leaf morphology (Kim et al., 2001), a dominant form of *GAI* mRNA resulted in modified leaf and fruit shapes (Haywood et al., 2005), a *BEL1-like* mRNA affected the tuberization (Banerjee et al., 2006), and dominant forms of two *Aux/IAA* mRNAs affected the lateral root formation (Notaguchi et al., 2012). In addition, many of phloem mobile mRNAs were identified in *Nicotiana benthamiana*/*Arabidopsis* hetero-grafting experiments, in which the derivatives of the *Arabidopsis* donor mRNAs were undoubtedly identified from recipient *N. benthamiana* scions by differences of their genome sequence information (Notaguchi et al., 2015). Although the transport mechanisms are still largely unknown, a few studies have provided information on RNA-binding proteins for each of small RNA and mRNA (Xoconostle-Cázares et al., 1999; Yoo et al., 2004; Ham et al., 2009) as well as the importance of 3D RNA structure in viral RNA movement (Takeda et al., 2011). Additionally, no detectable RNase activity has been observed in the phloem implying that RNA transport has important roles in plant physiology (Sasaki et al., 1998; Doering-Saad et al., 2002). However, direct evidence for the biological relevance of long-distance transport of mRNA is still missing.

These findings collectively suggested that the phloem translocation system is highly specialized for systemic signaling where a range of molecules are delivered as signal agents. Currently, physiological and developmental roles of only a few molecules have been characterized or identified. Future challenges are to investigate their roles of remains and to reveal their aspects of how each molecular species is used for systemic signaling.

## A Link of Xylem and Phloem Pathways

The necessity of long-distance signaling between separated organs, such as root-to-shoot, shoot-to-root, shoot-to-shoot and root-to-root, has been proposed through the observations of systemic responses to surrounding environmental conditions. For instance, plants respond to heterogeneous soil conditions of the availability of mineral macro- and micronutrients or local biotic stress such as insect attack, in which vascular tissues have been thought to be a pathway for the underlying long-distance signaling (Giehl et al., 2009; Liu et al., 2009; Soler et al., 2013). Xylem sap flow is directed from the roots to the shoot driven by water loss during transpiration and photosynthesis. In contrast, phloem sap flow is directed from the source mature leaves, where photosynthesis reaction is actively preceded with their large surfaces, to sink organs such as young developing meristems in the shoots and the roots. Therefore, translocations such as “root-to-shoot” and “shoot-to-root and shoot-to-shoot” can be simply achieved by the xylem and the phloem pathways, respectively.

On the other hand, “root-to-root” signaling, where signals are emitted from a part of roots and transmitted to another part of roots, could be partially explained by Ca^2+^ wave ranging in a part of roots (Choi et al., 2014); however, more generally, it appears to be achieved through the both xylem and phloem pathways, which has a “root-to-shoot-to-root” loop. This signaling circuit was more clearly established by recent discoveries of xylem mobile peptide signals as described in a previous section. The fact that receptors of xylem-mobile peptides are expressed in the phloem highlights a functional link between xylem and phloem signaling pathways. Analyses of GUS reporter lines for the receptors indicated that HAR1, a CLE-RS receptor, is preferentially expressed in the phloem (Nontachaiyapoom et al., 2007) and XIP1/CEPR1, a CEP1 receptor, is specifically expressed in the phloem (Bryan et al., 2012). These findings suggest that the phloem is the site where the root-derived peptides are converted to another secondary signal, and that such secondary messengers can be transported on the phloem sap flow toward “shoot-to-root.” In fact, in the CLE-RS/HAR1 cascade, CKs has been proposed as the shoot-to-root signals generated in the phloem to control nodulation. In the presence of CLE-RS peptides, HAR1 receptor promotes the production of shoot CKs through the up-regulation of a CK synthase, *LjIPT3*, in the phloem tissues; thus, the generated CKs could exert their activities to inhibit nodulation (Sasaki et al., 2014). In addition to nodulation control, the shoot-derived CKs could trigger the other physiological changes in the roots. Alternatively, other unrevealed secondary signal, if present, may explain this signaling specificity. Another important future question is the temporal aspects of shoot-derived CKs signaling to understand the mechanism to control the balanced symbiosis. Nodulation is a specialized phenomenon evolved in legumes that allows nitrogen fixation through a symbiosis with rhizobia, but the CKs biosynthesis is also strongly affected by nitrogen sources in plants other than legumes (Sakakibara, 2006). Hence, the CEP peptide pathway triggered by nitrogen starvation could also link with CKs cascade in downstream signaling.

These recent findings shed light on an advanced concept that the xylem and the phloem pathways develop a long-distance root-to-shoot and then shoot-to-root signaling feedback circuit in plants. In this scenario, signal molecules are transmitted across long-distances to response the soil environments via the vascular tissues with the following sequential processes. First, the information signaling molecules, generated in somewhere or in all parts of the branched root system, move shoot-ward via the xylem. Second, the signals run through a stem region between branched root and branched shoot and disperse to each of the mature leaves, possibly to the minor veins (**Figure 1A**). Third, the signal molecules are translocated from the xylem to the phloem and perceived by the receptors located on the phloem cells. In this process, the information is converted to the secondary signal inside of the phloem cells (**Figure 1B**). Fourth, the intracellular signal molecules travel on the phloem sap flow, including shoot-to-root translocation. Thus, finally the information signals generated in a part of root system can transmit to another part of the root. In each of these shoot- and root-ward translocation flows, all signaling molecules generated in branches of organs in response to heterologous environmental factors should be physically converged by running through a stem region (the bases of shoot and root), which ought to be the sole pathway (**Figure 1C**). This convergence might be the way to measure the entire signal level by averaging of the intensities of the signals generated in local and to make decisions how to respond or how much plants respond. Dosage control is a possible and quite likely mechanism that can be used to measure the level of signals. These points need to be addressed in the future studies. As described here, although plants do not have a circulatory system connecting the entire body as observed in animals, bi-directional signaling can be achieved by linking the xylem and phloem translocation pathways, and this may represent an elaborate signaling mechanism in plants.

## Concluding Remarks

In our current view, numerous mobile molecules that include secreted peptides in the xylem and proteins and RNA species in the phloem could have roles as specific information signals. In contrast to animal systems, where nervous systems and vascular tissues contribute to signaling and nutrient delivery (a sort of signaling as well), respectively, plant vascular systems serve as conduits for water and nutrients as well as long-distance signaling pathways which includes root-to-shoot signaling via the xylem, shoot-to-sink (such as the roots and young growing tissues in the shoots) signaling via the phloem, and a root-to-shoot-to-root circuit via the xylem as the first half and the phloem as the second half. Importantly, to reach their final destinations in each long-distance translocation pathway, short-distance transport after unloading usually at the terminus of vascular tissues is also necessary. The roles of most xylem peptides and phloem proteins and RNAs in plant development and physiology are still largely unknown. Additionally, long-distance transport has been suggested for the other molecules such as a lipid, glycerol-3-phosphate, that is used for systemic immunity (Chun et al., 2002; Chanda et al., 2011); however, their delivered pathway has yet to be elucidated. Reactive oxygen species may also serve as potential systemic signals (e.g., Miller et al., 2009). Thus, further studies are required to elucidate the functions and nature of vascular mobile molecules, together with the transport mechanisms involved in their movement.

## Conflict of Interest Statement

The authors declare that the research was conducted in the absence of any commercial or financial relationships that could be construed as a potential conflict of interest.

## Abbreviations

CC: companion cell
CEP: C-terminally encoded peptide
CK: cytokinin
CLE: CLV3/ESR-related
CLE-RS2: CLE-root signal 2
CLV: CLAVATAV
*CRA2*: *compact root architecture 2*
FT: FLOWERING LOCUS T
IAA: indole-3-acetic acid
*IPT*: *isopentenyltransferase*
LRR-RK: leucine-rich repeat receptor kinase
PD: plasmodesmata
SE: sieve element
TE: tracheary element
XIP1: xylem intermixed with phloem1
XSP10: xylem sap protein 10
